# Getting What Is Served? Feeding Ecology Influencing Parasite-Host Interactions in Invasive Round Goby *Neogobius melanostomus*

**DOI:** 10.1371/journal.pone.0109971

**Published:** 2014-10-22

**Authors:** Sebastian Emde, Judith Kochmann, Thomas Kuhn, Martin Plath, Sven Klimpel

**Affiliations:** 1 Institute for Ecology, Evolution and Diversity, Goethe-University, Frankfurt am Main, Hesse, Germany; 2 Senckenberg Gesellschaft für Naturforschung, Biodiversity and Climate Research Centre, Frankfurt am Main, Hesse, Germany; 3 College of Animal Science and Technology, Northwest Agriculture & Forestry University, Yangling, Shaanxi Province, P. R. China; Federal University of Viçosa, Brazil

## Abstract

Freshwater ecosystems are increasingly impacted by alien invasive species which have the potential to alter various ecological interactions like predator-prey and host-parasite relationships. Here, we simultaneously examined predator-prey interactions and parasitization patterns of the highly invasive round goby (*Neogobius melanostomus*) in the rivers Rhine and Main in Germany. A total of 350 *N. melanostomus* were sampled between June and October 2011. Gut content analysis revealed a broad prey spectrum, partly reflecting temporal and local differences in prey availability. For the major food type (amphipods), species compositions were determined. Amphipod fauna consisted entirely of non-native species and was dominated by *Dikerogammarus villosus* in the Main and *Echinogammarus trichiatus* in the Rhine. However, the availability of amphipod species in the field did not reflect their relative abundance in gut contents of *N. melanostomus*. Only two metazoan parasites, the nematode *Raphidascaris acus* and the acanthocephalan *Pomphorhynchus* sp., were isolated from *N. melanostomus* in all months, whereas unionid glochidia were only detected in June and October in fish from the Main. To analyse infection pathways, we examined 17,356 amphipods and found *Pomphorhynchus* sp. larvae only in *D. villosus* in the river Rhine at a prevalence of 0.15%. *Dikerogammarus villosus* represented the most important amphipod prey for *N. melanostomus* in both rivers but parasite intensities differed between rivers, suggesting that final hosts (large predatory fishes) may influence host-parasite dynamics of *N. melanostomus* in its introduced range.

## Introduction

Biological invasions have increased exponentially in recent years due to human activities, especially shipping, along with the adverse effects of environmental changes such as global warming [1]–[3]. Although brackish waters have the highest risk for species introductions, freshwater ecosystems are also strongly affected, especially by the introduction of non-indigenous fishes [4], [5]. Once established in their new environment, invasive non-indigenous species can have tremendous effects on local populations of indigenous species, e.g., through competitive [6], predator-prey [7]–[9], or host-parasite interactions [10], [11], all of which have the potential to result in altered ecosystem functioning (see review by Strayer [12]).

To date, several studies in aquatic ecosystems have considered the question of how invasive predators can affect native prey populations [13]–[15], or how invasive prey populations can alter indigenous prey communities [16], [17], and whether or not non-indigenous prey species become integrated into the prey spectrum of indigenous predators [18]. Furthermore, studies have started to concentrate on parasitization patterns of native and invasive species, and several different scenarios are possible: (i) invasive hosts may lose their original parasite load (‘enemy release hypothesis’), providing invasive species with an initial benefit in their novel range [10], [19], [20]. (ii) Introduced hosts may carry new parasite species (parasite spill-over), which may adversely affect native host species [21]. (iii) Invasive hosts may serve as intermediate hosts or vectors for local parasites or diseases (parasite spillback) [21]. (iv) Finally, shift and/or loss of local parasite species would be predicted if the invader is replacing local host species but cannot function as intermediate or definitive host in the parasite life cycles (dilution effect) [22], [23]. Few studies, however, have simultaneously considered predator-prey interactions and parasitization patterns of different trophic levels in ecosystems that are heavily influenced by invasive species [24]–[26]. This is surprising, given that many parasites with indirect life-cycles rely on the ingestion of their intermediate hosts by further (intermediate or final) host species to successfully complete their life cycles [27], [28]. Biological invasions could provide large numbers of host specimens within a very short time-span (e.g. [29]) that could affect parasite transmission patterns in entire fish communities.

The round goby *Neogobius melanostomus* (Pallas, 1814) is a frequent invader of brackish and freshwater habitats worldwide, reaching enormous population densities and causing changes of food web dynamics at different trophic levels, e.g., in the North American Great Lakes [30] and in large European rivers, e.g. the Danube [29]. Round gobies nowadays make up app. 80% of fish catches in the Rhine [31], and so an alteration of ecological interactions is also expected for the Rhine. For example, it is known that round gobies act as competitors of spawning or foraging sites with native species [30]. Feeding patterns of *N. melanostomus* vary in different distribution areas. While dreissenid mussels play an important role in the feeding ecology of *N. melanostomus* in the Great Lakes and in the Baltic Sea [32], [33], amphipods seem to be their main forage in German rivers [24], [25], [31]. In the Rhine, the Ponto-Caspian amphipod *Dikerogammarus villosus* (Sowinsky, 1894) has been described as dominating communities of macroinvertebrates and as an important prey species of *N. melanostomus* [24], [25], [31], [34], [35]. Both species, *D. villosus* and *N. melanostomus* function as intermediate hosts for different parasites (e.g., *Pomphorhynchus* spp. and *Raphidascaris* spp.) and may be responsible for the spread of these parasites, which could increasingly affect native vertebrate and invertebrate hosts as well [24], [36].

Studies on *N. melanostomus* that combine the analysis of their feeding habits with parasitological analyses are rare and have focused on the Danube [25], [29] and Rhine [24], [31]. To analyse the role of different amphipod species for metazoan fish parasite transmission as well as temporal variation of diet compositions in invasive *N. melanostomus*, samples from the rivers Main and Rhine were compared in this study. We hypothesized that (a) *N. melanostomus* will mainly feed on amphipods throughout the course of our repeated monthly sampling and in both rivers, and accordingly, (b) the availability of amphipod species in a given river will reflect their relative contribution to gut contents of *N. melanostomus*. Moreover, we expected that (c) monthly infestation rates of amphipods with parasite species and monthly feeding rates of amphipods by *N. melanostomus* should reflect parasite infestation rates in *N. melanostomus*. Finally, a detailed description of parasite fauna for two sampling locations in the rivers Main and Rhine was intended to complement current parasite diversity estimates of *N. melanostomus* in its introduced range.

## Materials and Methods

### Sampling

A total of *n* = 350 *N. melanostomus* were collected from June to October 2011 in the rivers Rhine (49°51′54.7″N 8°21′40.2″E) and Main (50°04′48.9″N 8°31′19.6″E) in Germany. Both sites were similar in habitat structure with rip-rap embanked shorelines (technolithal) that led into bottom substrate of sand and gravel. In contrast to the Rhine, the river bank of the Main had little more vegetation with roots partly reaching into the water.

35 *N. melanostomus* specimens per site were caught randomly on top of and around rip-raps (depths of ∼40–200 cm) during one day at the end of each month (between ∼9 am–2 pm) using a hook and line technique. Since standardized angling is known to yield an equilibrated sex ratio and homogenously distributed, relatively large-sized specimens in *N. melanostomus* [37], a fishing rod equipped with an anti-tangle bottom rig consisting of a special sinker (Tiroler Hölzl, 80 g) was used to avoid entanglement between rip-rap interstices. A small, round hook (Owner, barb special, size 14, FRL-044) was baited with 1–3 fly maggots. All hooked fish were used for subsequent examination in the laboratory without any size or sex selection. Each fish was carefully hooked off with a special hook removal tool and was humanely killed inside a plastic bag in order to avoid losing gut contents or parasites. To prevent further digestion or migration of parasites to other organs, fishes were kept separately in plastic bags in a cooling box filled with ice and stored afterwards at −20°C for later examination.

Amphipods were also collected monthly at the same sampling sites turning around large stones and using the ‘kick-sampling’ method after Storey et al. [38]. A small fishing net (15×20 cm, mesh size ∼1 mm) was used to catch as many amphipods as possible within 30 minutes along a 10 m stretch at a depth of up to 50 cm. Amphipods were kept together with organic material and some stones in plastic bags. Entire samples were frozen at −20°C and later separated from sediment to identify amphipods to species level.

### Parasitological examination and feeding ecology of *N. melanostomus*

Gobies were measured for total length (cm) and weight (g), condition factors (CF) were calculated according to Schäperclaus [39]. These measures are key parameters in studies on fish biology and were reported in (Text S1, Table S1) to facilitate comparisons with other studies.

Fish were then examined for their metazoan parasite fauna and stomach content using a stereomicroscope (Olympus SZ 61, magnification x 6.7–45). At first, skin, fins and gills were inspected for ectoparasites. Afterwards, the body cavity was opened to separate the inner organs. Body cavity, rinsed with 0.9% NaCl, gastrointestinal tract, gonads, kidney, liver, mesenteries, spleen and eyes were dissected and examined for endoparasites. Isolated parasites were freed from host tissue and preserved in 70% ethanol (with 4% glycerol) for morphological identification. To this end, glycerine preparations were made according to Riemann [40]. Determination under a microscope (Leitz Dialux 22, magnification x 15.75–630) was aided by original descriptions and descriptions of Golvan [41] and Špakulová et al. [42] for acanthocephalans, and Moravec [43] for nematodes. Subsamples were stored in 100% ethanol for genetic analysis (see Text S2).

Since gobies have no clearly separated stomach and a very short gut, the entire gastrointestinal tract was carefully cut lengthwise with a small pair of scissors. The weights of full and empty stomachs and the weights of each food item were recorded to the nearest 0.001 g after pat-drying on absorbent paper. Very small, as well as almost digested and defragmented parts of one prey group that could not be identified to species level were referred to as ‘not determined’ (indet.) and weighted as a pooled subsample. Only specimens that could clearly be identified, e.g. using assignable parts like eyes or telson, were identified and counted. Other components, mainly mucus and sand, but also undeterminable items were neglected. Prey organisms were sorted and identified to the lowest possible taxon and grouped into the following categories: amphipods, molluscs, insects and ‘others’ (plants, vertebrates, Acari). Isolated food organisms and parasites were preserved in 70% ethanol (with 4% glycerol) for morphological identification.

Amphipods were identified to species-level following Eggers & Martens [44], [45] and preserved in 70% ethanol. For parasitological examination, all amphipods were dissected and carefully screened under a stereomicroscope. Isolated parasites were stored in 100% ethanol. From each monthly sampling, fifty amphipods of each species were randomly taken to determine sex, body size and weight using an ocular micrometer and a micro-balance. Size was measured from the anterior rostrum to the base of the telson while animals were stretched in a straight position [46]. Data are reported in (Text S3, Figure S1).

### Statistical analyses

We first tested if the relative abundance of amphipods on site (covariate, arcsine(square root)-transformed percentages relative to the highest monthly abundance value observed for the respective site) determines the proportion of amphipods in *N. melanostomus* gut contents (monthly mean values were treated as the dependent variable) using analysis of covariance (ANCOVA using SPSS vs. 22), in which ‘site’ was a fixed factor. A Chi^2^ goodness-of-fit test (using R; R Development Core Team [47]) was then applied to test whether amphipod species compositions as encountered on site are reflected in gut contents.

Gut content analyses comprised calculations of the numerical percentage of prey (N%), the weight percentage of prey (W%), and the frequency of occurrence of prey (F%) [48], [49]. On the basis of these three indices, the index of relative importance (IRI) of different food items was calculated [50]. Differences in gut content assemblage structure between months and rivers were also assessed using two-factorial permutation ANOVA (PERMANOVA; 999 permutations) on Bray-Curtis dissimilarities of 4^th^-root transformed weights (mg) of the different species in each fish gut using the PRIMER v6 and PERMANOVA+ add-on package (PRIMER-e, Plymouth, UK). The SIMPER procedure [51] was used for post hoc identification of the source of variation.

Parasitological analyses comprised calculations of standard parameters: the prevalence (P), mean intensity (mI), intensity (I) and mean abundance (mA) for each parasite species according to Bush et al. [52]. High mean intensities of *Pomphorhynchus* sp. infections were found (see results), and previous studies suggested transmission pathways into *N. melanostomus* via amphipods, especially *D. villosus* [24]. Therefore, we used a repeated-measures General Linear Model (rmGLM using SPSS vs. 22) to test if mean intensities of *Pomphorhynchus* sp. in round gobies (dependent variable) differed between sexes (rm) and sites (fixed factor), and if the proportion of amphipods in the gut contents (arcsine(square root)-transformed numerical percentages, covariate) had an effect. The nematode *R. acus* was also relatively abundant in fish samples, but we restricted our analysis to non-parametric Wilcoxon signed-rank test (using SPSS vs. 22) to test whether differences in infection rates existed between the two rivers.

## Results

### Amphipod communities

**Figure 1:**
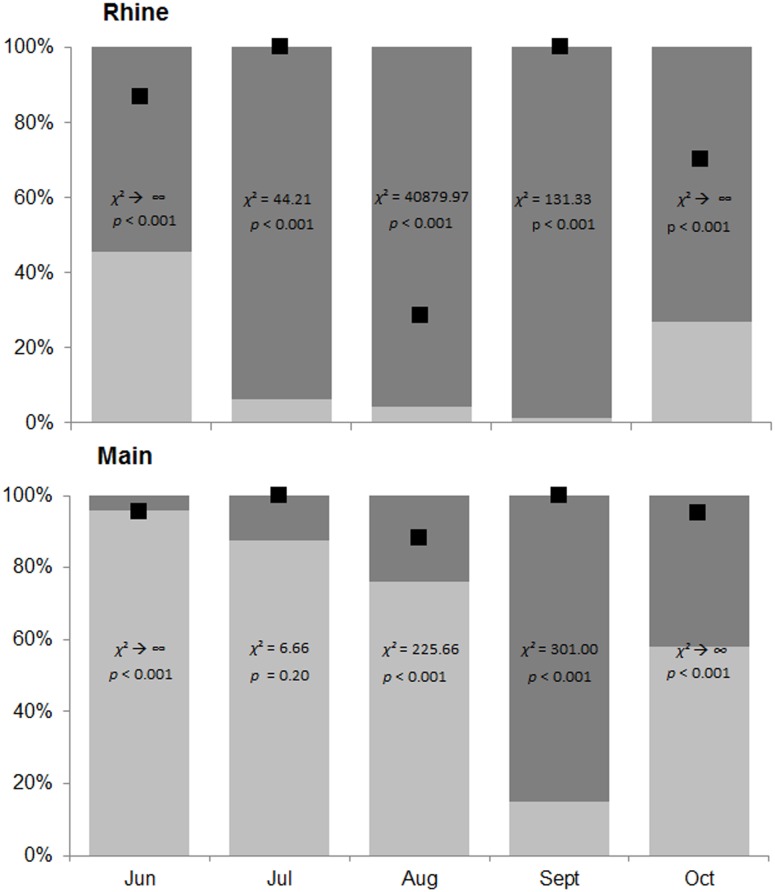
Dominant amphipod species. Fraction of the two dominant amphipod species (*D. villosus* = light grey, *E. trichiatus* = dark grey) in samples collected at our two study sites and numerical percentages of *D. villosus* in gut contents of *N. melanostomus* (black squares). Chi^2^ goodness-of-fit tests were used to compare the availability of different amphipod species on site (expected values) with observed compositions in gut contents. For total numbers of individuals and amphipod species see Table S2.

717 to 3,758 amphipods were collected during the monthly samplings, with a total of *n* = 9,820 in the Rhine and *n* = 7,536 in the Main (see Table S2). Five invasive but no native amphipod species were found in both rivers, namely *D. villosus*, *Echinogammarus trichiatus* (Martynov, 1932), *Echinogammarus ischnus* (Stebbing, 1899), *Chelicorophium curvispinum* (Sars, 1895) and *Chelicorophium robustum* (Sars, 1895). *Cryptorchestia cavimana* (Heller, 1865) occurred only in samples from the Main. *Dikerogammarus villosus* was dominating in all samples from the Main (total *n* = 5,346; 69%), except for September (Figure 1). In contrast, *E. trichiatus* was the dominant species in all samples from the Rhine (total *n* = 8,463; 86%; Figure 1). In both rivers a more balanced sex ratio was found for *D. villosus* (males:females, Rhine: 1∶1.03; Main: 1∶1.29) than for *E. trichiatus* (Rhine: 1∶2.36; Main: 1∶3.10).

### General feeding ecology of *N. melanostomus*

**Figure 2:**
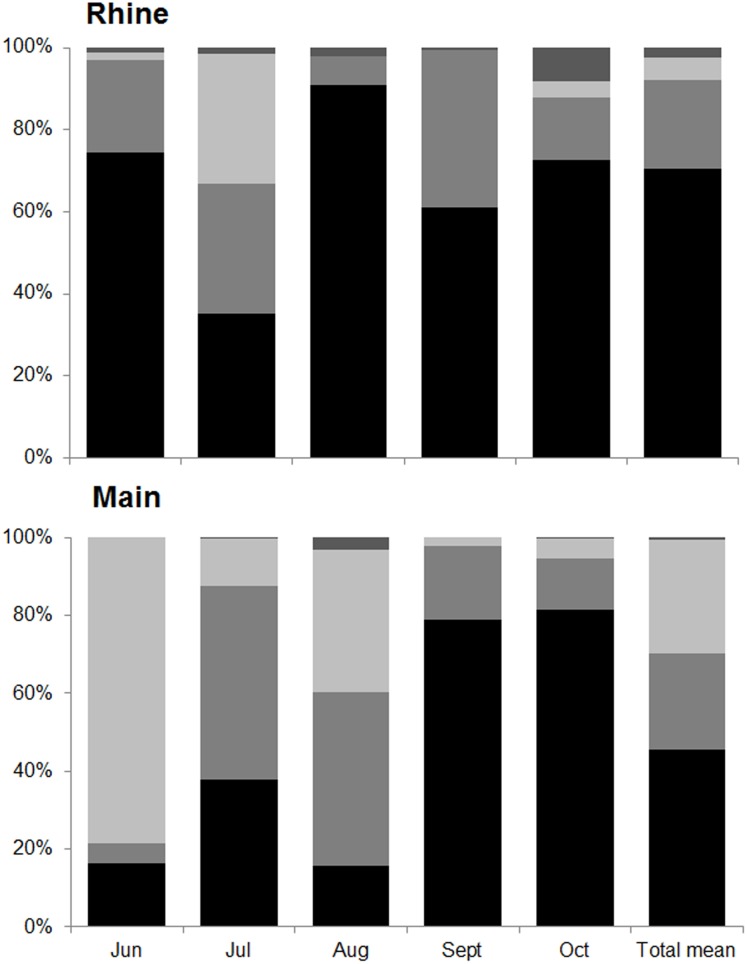
Gut contents of *Neogobius melanostomus*. Relative compositions (index of relative importance, IRI) of gut contents of *N. melanostomus* in two rivers from June until October 2011 as well as the total mean. Bar plot, from bottom to top: Amphipoda (black), Mollusca (medium grey), Insecta (light grey), others (dark grey).

18 (Rhine) and 16 (Main) different prey items were identified in *N. melanostomus* guts (Table S3, Table S4). The index of relative importance (IRI) found amphipods to be the main diet component of *N. melanostomus*, with an overall contribution of 71% in the Rhine and 46% in the Main (Figure 2). In the Rhine, amphipods contributed with at least 30% in each monthly sample (Figure 2). The second most important group was molluscs, which contributed with 7–38% to the overall gut content. The widespread and common species *Bithynia tentaculata, Potamopyrgus antipodarum* and *P. antipodarum f. carinata* were distinguishable, but, due to a high degree of fragmentation, were combined into ‘Gastropoda indet.’. Insects were rarely consumed, except for July where the IRI for Chironomidae rose to 2,288.83 (Table S3) when very little gut content was found overall. In the Main, highest proportions of amphipods (over 80%) occurred in September and October (Figure 2). Insects were consumed more often than in the Rhine, especially in June (79%) and August (36%). Fish diet was based on molluscs with 50% and 45% in July and August, respectively. Fishes, plants and Acari were rarely consumed in both rivers.

Gut content assemblage structure showed strong fluctuations between months and rivers. They differed significantly between June and July and June and August in the Rhine, whereas June and August were different from all other months in the Main (PERMANOVA: pseudo-*F* = 8.64, *df* = 4, *p* = 0.001 for the interaction ‘river × month’; for post hoc results see Table 1). Amphipods mostly accounted for the highest average dissimilarity between different monthly samples in the Rhine, whereas amphipods and insects accounted for the highest average dissimilarity between months in the Main (SIMPER procedure).

**Table 1:** PERMANOVA results (post hoc procedure).

River Rhine	*t*	*p* (perm)	River Main	*T*	*p* (perm)
**Jun, Jul**	2.842	0.001	Jun, Jul	5.006	0.001
**Jul, Aug**	3.310	0.001	Jun, Aug	3.155	0.001
			Jun, Sept	5.943	0.001
			Jun, Oct	3.758	0.001
			Jul, Aug	2.835	0.001
			Aug, Sept	4.269	0.001
			Aug, Oct	2.447	0.003

Differences in gut contents assemblage structure (based on species’ weights) of *N. melanostomus* between months. Significant results (permutation *p*<0.05) after Bonferroni correction for multiple comparisons are shown.

### Amphipod prey preference of *N. melanostomus*

Few individuals of *C. curvispinum* were found in *N. melanostomus* guts, and the dominating amphipod species was *D. villosus*, especially in the Main, but to a lesser degree also in the Rhine. This was reflected in the ANCOVA, which detected a significant interaction between ‘site’ and ‘relative abundance of amphipods on site’ (Table 2).

**Table 2:** ANCOVA results – all amphipods.

	Source	df	MS	*F*	*p*	Partial Etasquared
All amphipods	Site	1	0.317	9.330	**0.022**	0.609
	Rel. abundance	1	0.003	0.091	0.773	0.015
	Site × rel.abundance	1	0.549	16.183	**0.007**	0.730
	Residuals	6	0.034			

Numerical percentages of all amphipods in the gut content of *N. melanostomus* in relation to the relative abundance of amphipods on site. Significant effects are in bold.

**Figure 3:**
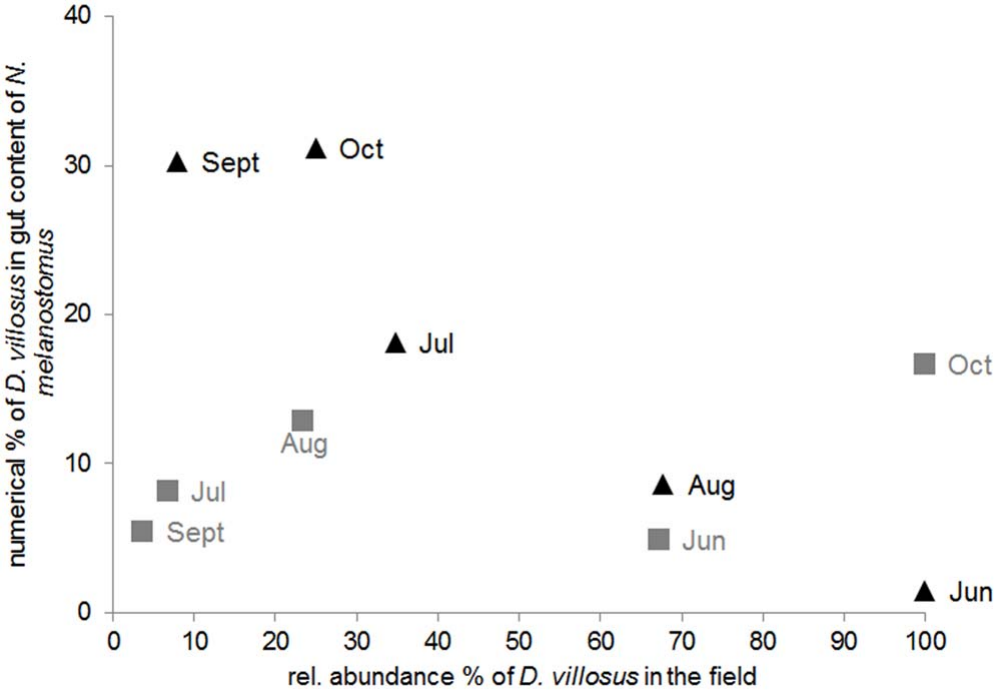
*Dikerogammarus villosus* in fish guts and in the field. Numerical percentage of *D. villosus* in gut contents of *N. melanostomus* in relation to the relative abundance of *D. villosus* at the Main (black) and Rhine (grey) between June and October 2011.

*Dikerogammarus villosus* was disproportionally frequent in gut contents given its availability relative to that of other amphipod species on site (Chi^2^ goodness-of-fit tests, *p*<0.001; except for the July sampling in the Main when *D. villosus* overall was highly abundant in the field; Figure 1). Therefore, an additional ANCOVA with similar model structure was run using percentages of *D. villosus* in the gut content of *N. melanostomus* as the dependent variable (Table 3). Whereas a decrease (not increase) of numerical percentages of *D. villosus* in the gut content of *N. melanostomus* with increasing availability of *D. villosus* on site was found in the Main (driving a significant main effect of the covariate; Table 3), this pattern was not observed in the river Rhine (see significant interaction effect in Table 3; Figure 3).

**Table 3:** ANCOVA results – *D. villosus*.

	Source	df	MS	*F*	*p*	Partial Etasquared
*D. villosus*	Site	1	0.155	31.261	**0.001**	0.839
	Rel. abundance	1	0.053	10.644	**0.017**	0.640
	Site × rel.abundance	1	0.127	25.487	**0.002**	0.809
	Residuals	6	0.005			

Numerical percentages of *D. villosus* in the gut content of *N. melanostomus* in relation to the relative abundance of *D. villosus* on site. Significant effects are in bold.

### Fish parasites: species identity and general biology

In total, three metazoan parasite species, two in the Rhine and three in the Main, could be isolated from *N. melanostomus*. The following taxa were identified morphologically: *Pomphorhynchus* sp., *Raphidascaris acus*, and Glochidia indet. (Table 4). As noted by Špakulová et al. [42] and Emde et al. [24], morphological identification of species within the acanthocephalan genus *Pomphorhynchus* can be difficult. Therefore, molecular barcoding was conducted on a subset of *n* = 3 specimens that were morphologically identified as *P. tereticollis.* Sequence data for ITS-1/5.8S/ITS-2 (Genbank accession numbers KJ756498–KJ756500) were almost identical (99.0% similarity, e-value: 0.00) to a sequence from *P. laevis* isolated from the cyprinid *Leuciscus cephalus* from the Czech Republic (Genbank accession number AY135415), suggesting that all acanthocephalan individuals in this study may belong to the same species. Due to a mismatch between the morphological identification characteristics and genetic information, acanthocephalan specimens were referred to as *Pomphorhynchus* sp. in this study.

**Table 4:** Parasitological parameters for the parasite fauna of *N. melanostomus*.

	P (%)					I_min_–I_max_/Mi					mA				
	Jun	Jul	Aug	Sept	Oct	Jun	Jul	Aug	Sept	Oct	Jun	Jul	Aug	Sept	Oct
***Rhine***															
**Nematoda**															
*Raphidascaris acus*(Cyst, L) in body cavity/mesentery/liver	50.00	28.57	57.14	55.56	34.29	1**–**6/2.44	1–5/2.10	1–18/3.80	1–17/3.67	1–7/10.25	1.22	0.60	2.17	1.89	3.51
**Acanthocephala**															
*Pomphorhynchus* sp. (L)in body cavity/mesentery/liver	86.11	71.43	100	100	74.29	1**–**118/22.65	1–35/9.04	1–101/24.26	2–95/32.71	1–20/8.46	19.50	6.46	24.26	32.71	6.29
**Total**	100	77.14	100	100	88.57	1**–**118/20.72	1–39/9.15	1–105/26.43	2–95/34.60	1–23/11.06	20.72	7.06	26.43	34.60	9.80
***Main***															
**Nematoda**															
*Raphidascaris acus*(Cyst, L) in body cavity/mesentery/liver	74.29	85.71	82.86	88.57	91.43	1**–**20/3.85	1–29/6.17	1–24/6.55	1–9/3.39	1–15/5.63	2.86	5.29	5.43	3.00	5.14
**Acanthocephala**															
*Pomphorhynchus* sp.(L) in body cavity/mesentery/liver	74.29	60.00	37.14	37.14	25.71	1–16/2.77	1–15/3.48	1–6/2.62	1–9/3.00	1–16/3.22	2.06	2.09	0.97	1.11	0.83
**Bivalvia**															
Glochidia indet. (L) in gills	54.29	–	–	–	38.10	1–8/3.68	–	–	–	1–23/9.88	2.00	–	–	–	3.76
**Total**	97.14	85.71	82.86	94.29	94.29	1–30/7.12	1–44/8.60	1–29/7.72	1–17/4.36	1–35/8.73	6.91	7.37	6.40	4.11	8.23
**Wilcoxon signed–rank test**															
*Raphidascaris acus*	z = –2.023 *p* = **0.043** (Rhine/Main)					z = –0.405 *p* = 0.686 (Rhine/Main)					z = –2.023 *p* = **0.043** (Rhine/Main)				
*Pomphorhynchus* sp.	z = –2.023 *p* = **0.042** (Rhine/Main)					z = –2.023 *p* = **0.043** (Rhine/Main)					z = –2.023 *p* = **0.043** (Rhine/Main)				

I = Intensity, L = larvae, mA = mean abundance, mI = mean intensity and P = prevalence.

All parasites were larval stages (Table 4). *Pomphorhynchus* sp. occurred only in the cystacanth stage. In the Rhine 91% of specimens were encysted in the mesenteries and liver and 9% were living freely in the body cavity. A similar pattern was found in the Main with 96% encysted in mesenteries and liver and 4% freely in the body cavity. The body cavity also harboured encysted *R. acus*, which occurred predominantly as L_2_-larvae (91% in the Main, 88% in the Rhine), and L_3_-larvae.

### Fish parasites: faunal composition

**Figure 4:**
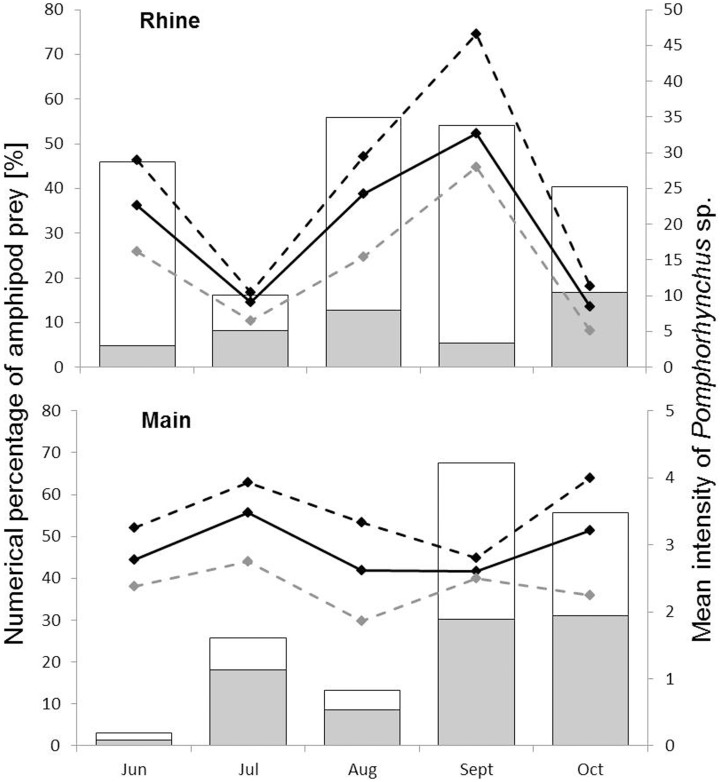
Amphipod prey and infections with *Pomphorhynchus* sp. Relationship between numerical percentages of *D. villosus* (grey) and Amphipoda indet. (white) in the gut content of *N. melanostomus* and mean intensities (mI, black line) of *Pomphorhynchus* sp. in male (grey dashed line) and female (black dashed line) *N. melanostomus.* For numbers of individuals please refer to Table S3 and Table S4.

The most prevalent metazoan parasite type was *Pomphorhynchus* sp. with 100% prevalence in August and September in fish caught in the Rhine (Table 4). Maximum intensity reached 118 specimens per fish. Highest prevalence of *Pomphorhynchus* sp. in the Main was recorded in June with 74.3%. Mean intensity of *Pomphorhynchus* sp. was an order of magnitude larger in fishes sampled from the Rhine (maximum mI = 34.6) than from the Main (maximum mI = 3.48) and always greater in female than in male *N. melanostomus* (rmGLM, significant interaction of ‘sex × site’; Table 5; Figure 4). The nematode *R. acus* occurred with significantly lower prevalence in the Rhine (min. 28.57%, max. 57.14%) than in the Main (74.29% and 91.43%; Wilcoxon signed-rank test, *z* = –2.023, *p* = 0.043; Table 4). A maximum intensity of specimens of *R. acus* per fish was detected. Undetermined glochidia, i.e., parasitic larvae of unionid bivalves were detected on fish gills only in June (P = 54.3%) and October (P = 38.1%) in the Main.

**Table 5:** Repeated–measures GLM results.

	Source	df	MS	*F*	*p*	Partial Eta squared
**Within–subjects effects**	Sex	1	35.935	3.689	0.096	0.345
	Sex × amphipods in gut	1	3.973	0.408	0.543	0.055
	Sex × site	1	129.212	13.263	**0.008**	0.655
	Residuals (Sex)	7	9.742			
**Between–subjects effects**	Intercept	1	510.007	3.202	0.117	0.314
	Amphipods in gut	1	46.853	0.294	0.604	0.040
	Site	1	1459.710	9.164	**0.019**	0.567
	Residuals	7	159.282			

Repeated–measures GLM on mean intensities of *Pomphorhynchus* sp. in round gobies in relation to fish sex, numerical percentages of amphipods (*D. villosus* and Amphipoda indet.) in the gut content and site. Significant effects are in bold.

### Parasites retrieved from amphipods

*Pomphorhynchus* sp. was the only parasite species that could be detected in amphipod samples. Two individuals were retrieved from *D. villosus* in the Rhine; the first was detected in samples from August (157 amphipods screened, P = 0.64%), the second in samples from October (671 amphipods screened, P = 0.15%). Overall, *Pomphorhynchus* sp. occurred at a prevalence of 0.15% in *D. villosus* in the river Rhine (two out of 1,350 specimens). The total number of *D. villosus* was four times larger in the Main than in the Rhine (i.e., *n* = 5,346), still, no parasites were detected. Low overall abundance precluded an analysis of potential temporal fluctuation in parasite infections of amphipods. Numerical percentages of amphipods in fish gut contents did not predict mean intensities of acanthocephalan parasites in *N. melanostomus* (Table 5).

## Discussion

### Feeding ecology of *N. melanostomus*

Co-evolved trophic relationships can facilitate biological invasions, as exemplified by communities of coexisting invasive *N. melanostomus*, dreissenid mussels and *E. ischnus* in the North American Great Lakes [53], [54]. Presence of co-evolved prey, however, appears not to be a prerequisite for *N. melanostomus* in German rivers, since *N. melanostomus* was characterized by an opportunistic and broad feeding strategy [see also 30,31]. Opportunistic feeding might also provide a plausible explanation for why we detected no positive correlation between the abundance of *D. villosus* in the field (generally a preferred type among amphipod prey) and their proportional contribution to gut contents. This was obvious especially during early summer, when prey species other than amphipods became more relevant (higher index of relative importance), especially in the Main, where insects and molluscs became the main food sources. Similarly, the importance of amphipod prey (*D. villosus* and others) for *N. melanostomus* in the Danube increased from early to late summer while the importance of chironomid larvae decreased [25]. Ingested insects in our present study were mostly nematoceran larvae, which are generally abundant in slow-flowing waterways like the Main. Non-biting midges (Chironomidae) no longer dominate the invertebrate community of the navigable main channel of the upper Rhine [55], which may explain why insects, overall, were barely ingested. While *N. melanostomus* is commonly regarded as a predator of fish eggs and fry (e.g. [56]), these were only rarely retrieved from gut contents.

An ontogenetic size dependent diet shift from amphipods and insects to a diet dominated mainly by molluscs is known for round gobies (e.g. [25]), however, fish lengths where shifts seem to occur vary substantially between study regions and most likely depend on availability and abundance of prey organisms [57], [58] as well as on time since invasion [29]. In our present study, the genus *Dreissena* seems to play a subordinate role compared to the Great Lakes and the Baltic Sea, which may be attributable to more readily available food sources, like insect larvae and amphipods. Generally, a tendency of increasing absolute numbers with increasing fish size was observed for *D. villosus* and nematocerans. In this context, Emde et al. [24] already demonstrated a size-dependent increase in acanthocephalan infections, which was inter alia explained by a correlation between goby and amphipod (*D. villosus*) prey body size, as it seems likely that the development of acanthocephalan larvae might only grow in amphipods above a certain size threshold. Thus, smaller gobies, feeding on smaller *D. villosus*, are less infected by acanthocephalans.

All amphipods found during monthly sampling were non-indigenous species from the Ponto-Caspian region (i.e., Black and Caspian Seas), corroborating studies in several European watersheds [24], [44], [59]. The most common non-indigenous amphipod species were *D. villosus* und *E. trichiatus*. *Dikerogammarus villosus* was dominant in samples from the Main, whereas *E. trichiatus* was dominant in Rhine samples, suggesting that faunal compositions of invasive amphipods may be more stable temporally and to a lesser degree spatially within the Rhine drainage (see also [24], [60]). *Dikerogammarus villosus* was detected six years earlier than *E. trichiatus* in the Rhine and is known for its strong predation on other gammarids [7], [61]. However, the total number of individuals caught in the Rhine was an order of magnitude lower than that of *E. trichiatus*. Whether higher predation on *D. villosus* by *N. melanostomus* in the Rhine compared to the Main could explain this pattern remains uncertain, since no fish densities at both sites were recorded herein.

Regardless of the high numbers of *E. trichiatus* in the Rhine, *N. melanostomus* primarily fed on *D. villosus*. How can this pattern be explained? Sih and Christensen [62] argued that variation in prey behaviour is more likely to affect the direction of predator-prey interactions than active prey choice of predators. Qualitatively, we noted that *E. trichiatus* at our study sites occurred closer to riverbanks, while *D. villosus* were found in both shallow and deeper waters, and so *E. trichiatus* could avoid fish predation in shallower habitats or by hiding between rip-rap interstices. Spatial niche segregation between *E. trichiatus* and *D. villosus* was previously observed in the Netherlands where the former seems to occur on soft substrates whereas the latter is most abundant on hard substrates [63]. Thus, different microhabitat use or different activity patterns in *D. villosus* are likely explanations for their dominance among amphipod prey in *N. melanostomus*.

Parasites can manipulate the predator avoidance of freshwater amphipods, rendering them more vulnerable to their fish predators (for *Gammarus pulex* see [64], [65]). Whether infections by *Pomphorhynchus* sp. affect the predator avoidance of *D. villosus* is currently not known, but if infected individuals were indeed more prone to predation, this would provide a striking explanation for our finding that gobies were highly infected by *Pomphorhynchus* sp., yet infectious stages were barely found in their amphipod prey (i.e., *D. villosus*), and were even completely absent in the Main. It seems reasonable to argue that infected *D. villosus* were ingested at an accelerated rate compared to uninfected specimens. Generally, infection rates of invertebrate intermediate hosts, especially crustaceans, tend to be low, often ranging between 0.01 and 0.1% prevalence [23], [28]. A possible reason for the higher infestation rates of *D. villosus* in the Rhine might be the presence of more final hosts (like common barbel *Barbus barbus* and European chub *Squalius cephalus,* however this assumption is not based on quantitative data but on personal observations only (S. Emde, personal observation).

*Pomphorhynchus* sp. is known to include a variety of different first intermediate hosts in its life cycle, such as *D. villosus* [24], *G. pulex* [64] and *C. curvispinum* [65]. *Gammarus pulex* seems to be completely displaced by invasive species in the Rhine and Main [24] and was not part of the gobies’ diet at both sampling sites. Following a massive decrease since 1995, *C. curvispinum* currently also plays a negligible role in the gobies’ diet [65]. In the light of the decrease of other amphipod species and the observed dominance of *D. villosus* in the gobies’ diet, we suggest that *D. villosus* currently represents the most relevant intermediate host for *Pomphorhynchus* sp. Still, future studies could investigate additional invertebrate groups and might uncover additional first intermediate hosts for the opportunistic parasites of the genus *Pomphorhynchus*.

### Parasite fauna of *N. melanostomus*

More than 94 parasites of *N. melanostomus* have been recorded worldwide [66], and in its introduced range in Europe, 35 metazoan parasite species have been detected so far (e.g., [66]–[69]). *Neogobius melanostomus* usually carries more than ten different parasite species per population in its native range [70]. Herein, only three parasite species could be detected in 350 round gobies examined, suggesting that the diversity of *N. melanostomus* parasites in the Rhine did not change over the past four years ([24], S. Emde personal observation). In other regions, the parasite fauna of invasive *N. melanostomus* increased rapidly, e.g., in the Gulf of Dansk, where numbers rose from 4 to 12 parasite species within two years [68]. Only 6 to 10 years have passed since round gobies were first recorded in German inland waterways, while the first report of round gobies at our sampling sites was in 2007 [71], [72]. Our results support the ‘enemy release hypothesis’ [19], and release from natural parasites could be one reason promoting the fast spread of round gobies worldwide. This advantage over indigenous fishes, however, will likely be lost if the diversity of the parasite fauna of *N. melanostomus* increases with time [73]. Whether or not such an increase of parasite diversity will occur in the future requires further monitoring.

All parasites detected in *N. melanostomus* were larval stages, and so we tentatively argue that currently no native parasite species is able to use *N. melanostomus* as its final but only as a paratenic host. A higher parasitization of *N. melanostomus* was observed in the Rhine, where fishes were also smaller and had a lower condition factor than in the Main (Text S1, Table S1). A high parasite load can lead to decreased growth in their fish hosts [74], however, infection studies in controlled environments would be needed to further address this hypothesis.

*Pomphorhynchus* sp. (Acanthocephala) and *Raphidascaris acus* (Nematoda) have been detected before in *N. melanostomus* caught in the Rhine, with similar infection rates for *Pomphorhynchus* sp. [24]. Latest data of the Danube River also described high abundances of this parasite but detected highest abundances in more recently invaded areas [29]. Similarly high prevalences of *R. acus* as found in our current study (up to 91.43%) are known from studies in other sections of the Rhine (56%) [75] and the Danube (P = 57%) [67]. Generally, differences in infection rates (prevalence/intensities) among studies could be related to the presence/absence as well as abundance of the parasites’ final hosts. For adult *R. acus* the European pike (*Esox lucius*) and brown trout (*Salmo trutta fario*) are known as principal final hosts [43], whereas it is barbel (*Barbus barbus*) and chub (*Squalius cephalus*) for *Pomphorhynchus* sp. [24]. However, *N. melanostomus* seems to represent a new, additional intermediate host for these parasites and thus, bridges the trophic level towards new potential, predatory final hosts. Other potential definitive hosts in the rivers Rhine and Main are trout (*Salmo trutta*) and catfish (*Silurus glanis*) for *Pomphorhynchus* [76], [77] and the European eel (*Anguilla anguilla*), European perch (*Perca fluviatilis*) and pike-perch (*Sander lucioperca*) for *R. acus* [78]. Infection studies need to show whether the female parasite attains gravidity in the potential definitive host or whether these predatory fishes may only act as para-definitive hosts in which the parasite matures but is unable to produce eggs [78]. If they do not act as definitive hosts, the large number of parasite larvae in *N. melanostomus* will be transmitted to these predatory fishes, however, not be able to complete their life cycle. This would lead to a dilution effect, resulting in a continued loss of infection within the system as has been described for different parasite-host communities before [79], [80] and would therefore be an alternative plausible explanation for the lower infection rates in the Main than in the Rhine.

Parasitic larval stages (Glochidia) of freshwater mussels of the family Unionidae were found in samples from the river Main, which confirms a former report of *N. melanostomus* serving as a host for unionid glochidia in the Danube [67]. Glochidia could be detected only during some months, because river mussels (*Unio* sp.) spawn in early summer and swan mussels (*Anodonta* sp.) in late summer, and glochidia attach to fish gills for only a few weeks [81]. Although unionid mussels are known to occur in the Rhine [82], no glochidia were detected on the gills of *N. melanostomus*, which could suggest an abundance-correlated effect. Alternatively, *N. melanostomus* might be a bad host for unionids [83]. Authors infected gobies with Glochidia of which 98% were lost within 16 days. Based on that study, our findings of Glochidia attached to gills of *N. melanostomus* could therefore be a finding that was the result of a very recent infection.

We initially hypothesized monthly infestation rates of *D. villosus* with *Pomphorhynchus* sp. potentially reflecting infestation rates in *N. melanostomus*. Due to overall low abundances of *Pomphorhynchus* sp. in *D. villosus* a statistical analysis in this direction was not possible. We also tested whether the numerical percentage of *D. villosus* in gut contents predicts mean intensities of *Pomphorhynchus* sp. but found no such effect. The timing of the parasite’s life cycle, however, has not yet been examined, and so our analysis (that was based on monthly sampling) may not have been appropriate to capture such potential effect.

Sex-related differences in parasite infections are common and can be ascribed to sex-specific behavioural, physiological or morphological differences [84], [85]. In this study, mean intensity of *Pomphorhynchus* sp. was significantly higher in females than males in the Rhine, supporting the finding of Brandner et al. [29] from the Danube River. No significant sex differences were observed in the Main, but *Pomphorhynchus* sp. mean intensities were low in the Main overall. Males can allocate much less time to feeding than females (for poeciliid fishes see [86], [87]) lowering their risk to take up parasites from food. Indeed, Charlebois et al. [88] found *N. melanostomus* males to cease feeding during brood care, while females producing eggs should have increased energy demands.

Our study confirmed that *D. villosus* functions as the main amphipod prey species for *N. melanostomus* in German rivers, however, parasite intensities in *N. melanostomus* differed between sampling locations of Rhine and Main independently of amphipod abundances. We suggest that a characterization of new final fish hosts, especially for *Pomphorhynchus* sp., at the sites investigated herein could provide important new insight into the ecological causes of variation in parasitization patterns of *N. melanostomus* in its introduced range.

## Supporting Information

Figure S1 **Box–plots of total length and total weight of two amphipod species.**
(TIF)

Table S1 **Biological parameters of** ***Neogobius melanostomus*** **.**
(DOCX)

Table S2 **Amphipod fauna.**
(DOCX)

Table S3 **Gut contents and parameters of** ***Neogobius melanostomus*** **for the river Rhine.**
(DOC)

Table S4 **Gut contents and parameters of** ***Neogobius melanostomus*** **for the river Main.**
(DOC)

Text S1 **Size measurements and condition factors of** ***N. melanostomus*** **.**
(DOCX)

Text S2 **Genetic identification of parasites.**
(DOCX)

Text S3 **Size measurements of** ***D. villosus*** **and** ***E. trichiatus.***
(DOCX)
