# Supplementary figures and images for: Getting What Is Served? Feeding Ecology Influencing Parasite-Host Interactions in Invasive Round Goby Neogobius melanostomus
Source: PLoS One. 2014 Oct 22;9(10):e109971. doi: 10.1371/journal.pone.0109971 (PMC4206283; doi:10.1371/journal.pone.0109971)

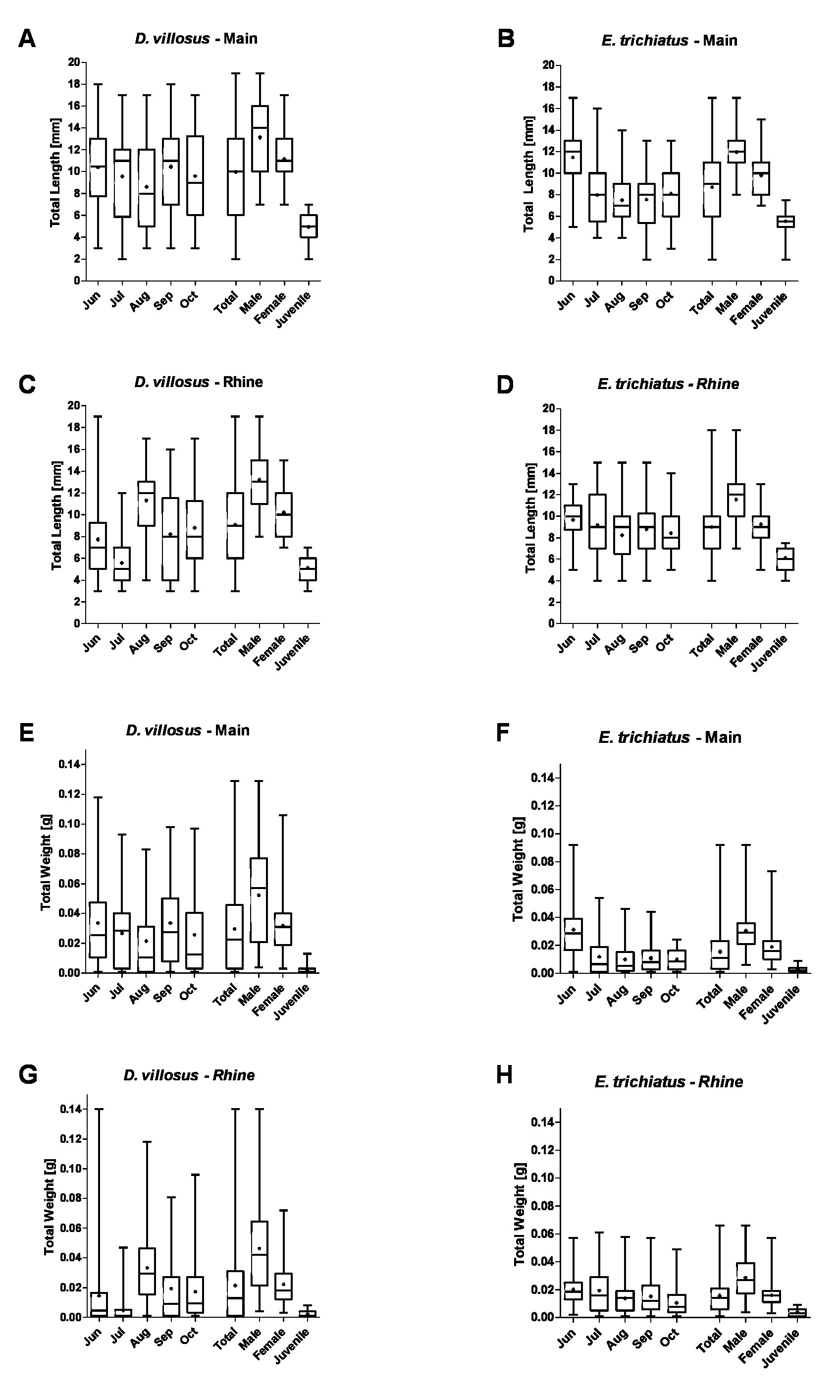

Supplement: Figure S1 — Box–plots of total length and total weight of two amphipod species. (TIF) [file pone.0109971.s001.tif]
