# Supplementary material for: Getting What Is Served? Feeding Ecology Influencing Parasite-Host Interactions in Invasive Round Goby Neogobius melanostomus
Source: PLoS One. 2014 Oct 22;9(10):e109971. doi: 10.1371/journal.pone.0109971 (PMC4206283; doi:10.1371/journal.pone.0109971)
Supplement: Table S1 — Biological parameters of Neogobius melanostomus . (DOCX) [file pone.0109971.s002.docx]

**Table S1.** **Biological parameters of Neogobius *melanostomus*.**

|  | **STL** | **TL** | **CF (± SD)** | **TW** | **SW** |
| --- | --- | --- | --- | --- | --- |
| **Rhine** |  |  |  |  |  |
| Jun | 8.41 | 9.74 | 1.56 (0.23) | 15.31 | 13.56 |
| Jul | 7.64 | 8.94 | 1.27 (0.08) | 10.71 | 9.68 |
| Aug | 8.17 | 9.61 | 1.22 (0.10) | 12.37 | 11.45 |
| Sept | 9.28 | 10.91 | 1.49 (0.23) | 21.63 | 19.60 |
| Oct | 6.90 | 8.16 | 1.43 (0.15) | 8.52 | 7.77 |
| **Main** |  |  |  |  |  |
| Jun | 9.21 | 10.77 | 1.77 (0.17) | 23.51 | 20.34 |
| Jul | 8.85 | 10.35 | 1.42 (0.11) | 20.13 | 17.83 |
| Aug | 8.71 | 10.17 | 1.35 (0.12) | 17.80 | 16.32 |
| Sept | 8.45 | 9.84 | 1.43 (0.12) | 15.56 | 14.15 |
| Oct | 7.74 | 9.06 | 1.38 (0.12) | 11.70 | 10.77 |

Shown are means of n = 35 individuals per sampling point. STL = standard length, TL = total length, CF = condition factor, TW = total weight, SW = slaughtering weight
