# Supplementary material for: Getting What Is Served? Feeding Ecology Influencing Parasite-Host Interactions in Invasive Round Goby Neogobius melanostomus
Source: PLoS One. 2014 Oct 22;9(10):e109971. doi: 10.1371/journal.pone.0109971 (PMC4206283; doi:10.1371/journal.pone.0109971)
Supplement: Table S2 — Amphipod fauna. (DOCX) [file pone.0109971.s003.docx]

**Table S2. Amphipod fauna.**

|  | Jun | | Jul | | Aug | | Sept | | Oct | | Total | |
| --- | --- | --- | --- | --- | --- | --- | --- | --- | --- | --- | --- | --- |
|  | R | M | R | M | R | M | R | M | R | M | R | M |
| **Amphipoda (n)** | **988** | **2366** | **717** | **908** | **3758** | **2034** | **1871** | **1236** | **2486** | **992** | **9820** | **7536** |
| *Dikerogammarus villosus* | 451 | 2266 | 46 | 791 | 157 | 1537 | 25 | 182 | 671 | 570 | 1350 | 5346 |
| *Echinogammarus trichiatus* | 537 | 100 | 670 | 113 | 3598 | 487 | 1843 | 1032 | 1815 | 413 | 8463 | 2145 |
| *Echinogammarus ischnus* | - | - | 1 | 2 | - | 6 | 3 | 7 | - | 9 | 4 | 24 |
| *Chelicorophium. curvispinum* | - | - | - | 1 | 2 | 3 | - | - | - | - | 2 | 4 |
| *Chelicorophium. robustum* | - | - | - | 1 | 1 | 1 | - | - | - | - | 1 | 2 |
| *Cryptorchestia cavimana* | - | - | - | - | - | - | - | 15 | - | - | - | 15 |

Amphipod fauna in the river Rhine (R) and Main (M) sampled over five months in 2011**.**
