# Supplementary material for: Getting What Is Served? Feeding Ecology Influencing Parasite-Host Interactions in Invasive Round Goby Neogobius melanostomus
Source: PLoS One. 2014 Oct 22;9(10):e109971. doi: 10.1371/journal.pone.0109971 (PMC4206283; doi:10.1371/journal.pone.0109971)
Supplement: Table S3 — Gut contents and parameters of Neogobius melanostomus for the river Rhine. (DOC) [file pone.0109971.s004.doc]

**Table S3.** **Gut contents of *Neogobius melanostomus* and calculated parameters for the river Rhine.**

| **Ecological Parameters** | Month | **MOLLUSCA** | Bivalvia  *Dreissena sp.* | Bivalvia  *Sphaerium solidum* | Bivalvia  *Sphaerium corneum* | Gastropoda  *Ancylus fluviatilis* | Gastropoda  *Theodoxus sp.* | Gastropoda indet. | **CRUSTACEA** | Amphipoda  *Chelicorophium curvispinum* | Amphipoda  *Dikerogammarus villosus* | Amphipoda indet. | **INSECTA** | Chironimidae | Nematocera indet. | Odonata | **ACARI**  Hydrachnidiae | **Others** | Plantea | Pisces |
| --- | --- | --- | --- | --- | --- | --- | --- | --- | --- | --- | --- | --- | --- | --- | --- | --- | --- | --- | --- | --- |
|  |  |  |  |  |  |  |  |  |  |  |  |  |  |  |  |  |  |  |  |  |
| **n** | Jun | **119** | 1 | 6 | 2 | 1 | - | 109 | **124** | 2 | 13 | 109 | **20** | - | 19 | 1 | **-** | **3** | 1 | 2 |
|  | Jul | **9** | - | - | 2 | - | - | 7 | **6** | - | 3 | 3 | **20** | 20 | - | - | **1** | **1** | - | 1 |
|  | Aug | **10** | 6 | 1 | 2 | - | - | 1 | **96** | 35 | 14 | 47 | **-** | - | - | - | **-** | **3** | 1 | 2 |
|  | Sept | **15** | 3 | - | - | - | - | 12 | **20** | - | 2 | 18 | **1** | 1 | - | - | **-** | **1** | 1 | - |
|  | Oct | **10** | 1 | - | 4 | - | 3 | 2 | **20** | 3 | 7 | 10 | **9** | 9 | - | - | **-** | **3** | 2 | 1 |
| **F%** | Jun | **60.61** | 3.03 | 9.09 | 6.06 | 3.03 | - | 48.48 | **96.97** | 3.03 | 9.09 | 84.85 | **27.27** | - | 24.24 | 3.03 | **-** | **9.09** | 3.03 | 6.06 |
|  | Jul | **40.00** | - | - | 13.33 | - | - | 33.33 | **33.33** | - | 13.33 | 20.00 | **40.00** | 40.00 | - | - | **6.67** | **6.67** | - | 6.67 |
|  | Aug | **25.81** | 16.13 | 3.23 | 6.45 | - | - | 3.23 | **90.32** | 32.26 | 16.13 | 48.39 | **-** | - | - | - | **-** | **9.68** | 3.23 | 6.45 |
|  | Sept | **38.89** | 16.67 | - | - | - | - | 22.22 | **66.67** | - | 5.56 | 61.11 | **5.56** | 5.56 | - | - | **-** | **5.56** | 5.56 | - |
|  | Oct | **33.33** | 5.56 | - | 11.11 | - | 11.11 | 5.56 | **72.22** | 11.11 | 16.67 | 44.44 | **16.67** | 16.67 | - | - | **-** | **16.67** | 11.11 | 5.56 |
| **W%** | Jun | **10.47** | 0.93 | 2.36 | 0.93 | 0.07 | - | 6.18 | **68.24** | 0.07 | 13.08 | 55.09 | **2.78** | - | 0.46 | 2.32 | **-** | **18.51** | 12.33 | 6.18 |
|  | Jul | **33.33** | - | - | 14.33 | - | - | 19.00 | **60.33** | - | 49.00 | 11.33 | **3.17** | 3.17 | - | - | **0.17** | **3.00** | - | 3.00 |
|  | Aug | **28.22** | 19.99 | 4.52 | 2.68 | - | - | 1.03 | **46.94** | 11.69 | 14.41 | 20.84 | **-** | - | - | - | **-** | **24.85** | 0.59 | 24.26 |
|  | Sept | **57.13** | 28.20 | - | - | - | - | 28.93 | **36.94** | - | 8.98 | 27.96 | **0.06** | 0.06 | - | - | **-** | **5.88** | 5.88 | - |
|  | Oct | **18.08** | 1.21 | - | 11.62 | - | 4.24 | 1.01 | **44.14** | 0.91 | 24.04 | 19.19 | **0.91** | 0.91 | - | - | **-** | **36.87** | 25.96 | 10.91 |
| **N%** | Jun | **44.75** | 0.38 | 2.26 | 0.75 | 0.38 | - | 40.98 | **46.62** | 0.75 | 4.89 | 40.98 | **7.52** | - | 7.14 | 0.38 | **-** | **1.13** | 0.38 | 0.75 |
|  | Jul | **24.33** | - | - | 5.41 | - | - | 18.92 | **16.22** | - | 8.11 | 8.11 | **54.05** | 54.05 | - | - | **2.70** | **2.70** | - | 2.70 |
|  | Aug | **9.17** | 5.50 | 0.92 | 1.83 | - | - | 0.92 | **88.07** | 32.11 | 12.84 | 43.12 | **-** | - | - | - | **-** | **2.75** | 0.92 | 1.83 |
|  | Sept | **40.54** | 8.11 | - | - | - | - | 32.43 | **54.06** | - | 5.41 | 48.65 | **2.70** | 2.70 | - | - | **-** | **2.70** | 2.70 | - |
|  | Oct | **23.80** | 2.38 | - | 9.52 | - | 7.14 | 4.76 | **47.62** | 7.14 | 16.67 | 23.81 | **21.43** | 21.43 | - | - | **-** | **7.14** | 4.76 | 2.38 |
| **IRI** | Jun | **3345.75** | 3.95 | 41.94 | 10.19 | 1.36 | - | 2286.46 | **11137.47** | 2.49 | 163.30 | 8151.27 | **281.06** | - | 184.42 | 8.18 | **-** | **178.49** | 38.49 | 42.02 |
|  | Jul | **2306.31** | - | - | 263.18 | - | - | 1263.96 | **2551.65** | - | 761.44 | 388.83 | **2288.83** | 2288.83 | - | - | **19.13** | **38.02** | - | 38.02 |
|  | Aug | **965.14** | 411.25 | 17.54 | 29.15 | - | - | 6.28 | **12193.97** | 1412.81 | 439.52 | 3094.70 | **-** | - | - | - | **-** | **267.06** | 4.86 | 168.33 |
|  | Sept | **3798.12** | 605.12 | - | - | - | - | 1363.53 | **6065.70** | - | 79.89 | 4681.42 | **15.35** | 15.35 | - | - | **-** | **47.69** | 47.69 | - |
|  | Oct | **1396.34** | 19.96 | - | 234.89 | - | 126.50 | 32.07 | **6627.14** | 89.47 | 678.45 | 1911.18 | **372.29** | 372.29 | - | - | **-** | **733.53** | 341.35 | 73.83 |

F = "frequency of occurrence", IRI = "index of relative importance", N = "numerical percentage of prey", n = "number of prey organisms", and W = "weight percentage of prey“
