# Supplementary material for: Getting What Is Served? Feeding Ecology Influencing Parasite-Host Interactions in Invasive Round Goby Neogobius melanostomus
Source: PLoS One. 2014 Oct 22;9(10):e109971. doi: 10.1371/journal.pone.0109971 (PMC4206283; doi:10.1371/journal.pone.0109971)
Supplement: Table S4 — Gut contents and parameters of Neogobius melanostomus for the river Main. (DOC) [file pone.0109971.s005.doc]

**Table S4. Gut contents of *Neogobius melanostomus* and calculated parameters for the river Main.**

| **Ecological Parameters** | Month | **MOLLUSCA** | Bivalvia  *Corbicula sp.* | Bivalvia  *Dreissena sp.* | Bivalvia  *Sphaerium solidum* | Bivalvia  *Sphaerium corneum* | Gastropoda  *Ancylus fluviatilis* | Gastropoda indet. | **CRUSTACEA** | Amphipoda  *Chelicorophium curvispinum* | Amphipoda  *Dikerogammarus villosus* | Amphipoda indet. | **INSECTA** | Chironimidae | Insecta indet. | Psychodidae | Nematocera indet. | **COLEOPTERA**  Coleoptera larva | **ACARI**  Hydrachnidiae | **OTHERS** | Plantea | Pisces |
| --- | --- | --- | --- | --- | --- | --- | --- | --- | --- | --- | --- | --- | --- | --- | --- | --- | --- | --- | --- | --- | --- | --- |
|  |  |  |  |  |  |  |  |  |  |  |  |  |  |  |  |  |  |  |  |  |  |  |
| **n** | Jun | **48** | - | 1 | 1 | 10 | - | 36 | **85** | 2 | 40 | 43 | **2661** | - | - | - | 2661 | **-** | **-** | **2** | 1 | 1 |
|  | Jul | **74** | - | 3 | 20 | 3 | - | 48 | **64** | - | 45 | 19 | **108** | 108 | - | - | - | **-** | **-** | **3** | 2 | 1 |
|  | Aug | **66** | 1 | - | 8 | 1 | 1 | 55 | **37** | 3 | 22 | 12 | **141** | 120 | 18 | 3 | - | **1** | **2** | **10** | 9 | 1 |
|  | Sept | **35** | - | 1 | 2 | 9 | 1 | 22 | **116** | - | 52 | 64 | **19** | 19 | - | - | - | **-** | **-** | **2** | 2 | - |
|  | Oct | **13** | - | - | 6 | 2 | - | 5 | **35** | 1 | 19 | 15 | **12** | 12 | - | - | - | **-** | **-** | **1** | 1 | - |
| **F%** | Jun | **57.14** | - | 2.86 | 2.86 | 22.86 | - | 45.71 | **71.43** | 2.86 | 31.43 | 40.00 | **97.14** | - | - | - | 97.14 | **-** | **-** | **5.71** | 2.86 | 2.86 |
|  | Jul | **82.14** | - | 10.71 | 39.29 | 7.14 | - | 50.00 | **82.14** | - | 57.14 | 25.00 | **39.29** | 39.29 | - | - | - | **-** | **-** | **10.71** | 7.14 | 3.57 |
|  | Aug | **57.14** | 2.86 | - | 17.14 | 2.86 | 2.86 | 42.86 | **40.00** | 2.86 | 20.00 | 20.00 | **62.86** | 54.29 | 22.86 | 2.86 | - | **2.86** | **5.71** | **25.71** | 22.86 | 2.86 |
|  | Sept | **51.43** | - | 2.86 | 2.86 | 14.29 | 2.86 | 31.43 | **91.43** | - | 37.14 | 54.29 | **25.71** | 25.71 | - | - | - | **-** | **-** | **5.71** | 5.71 | - |
|  | Oct | **35.0** | - | - | 10.00 | 10.00 | - | 20.00 | **75.00** | 5.00 | 35.00 | 35.00 | **30.00** | 30.00 | - | - | - | **-** | **-** | **5.00** | 5.00 | - |
| **W%** | Jun | **13.73** | - | 0.10 | 0.83 | 1.51 | - | 11.29 | **37.62** | 0.05 | 18.74 | 18.83 | **47.56** | - | - | - | 47.56 | **-** | **-** | **1.08** | 0.35 | 0.73 |
|  | Jul | **57.01** | - | 1.67 | 26.52 | 0.27 | - | 28.55 | **40.04** | - | 34.20 | 5.84 | **0.71** | 0.71 | - | - | - | **-** | **-** | **2.24** | 0.95 | 1.29 |
|  | Aug | **57.58** | 12.57 | - | 8.97 | 0.03 | 0.09 | 35.92 | **26.98** | 0.31 | 21.82 | 4.85 | **6.55** | 3.78 | 2.56 | 0.21 | - | **0.06** | **0.12** | **8.70** | 8.06 | 0.64 |
|  | Sept | **35.94** | - | 1.19 | 7.03 | 23.66 | 0.11 | 3.95 | **62.73** | - | 34.57 | 28.16 | **0.58** | 0.58 | - | - | - | **-** | **-** | **0.74** | 0.74 | - |
|  | Oct | **24.54** | - | - | 3.41 | 10.39 | - | 10.74 | **72.81** | 0.12 | 60.16 | 12.53 | **0.92** | 0.92 | - | - | - | **-** | **-** | **1.73** | 1.73 | - |
| **N%** | Jun | **1.73** | - | 0.04 | 0.04 | 0.36 | - | 1.29 | **3.04** | 0.07 | 1.43 | 1.54 | **95.17** | - | - | - | 95.17 | **-** | **-** | **0.08** | 0.04 | 0.04 |
|  | Jul | **29.71** | - | 1.20 | 8.03 | 1.20 | - | 19.28 | **25.70** | - | 18.07 | 7.63 | **43.37** | 43.37 | - | - | - | **-** | **-** | **1.20** | 0.80 | 0.40 |
|  | Aug | **25.68** | 0.39 | - | 3.11 | 0.39 | 0.39 | 21.4 | **14.40** | 1.17 | 8.56 | 4.67 | **54.86** | 46.69 | 7.00 | 1.17 | - | **0.39** | **0.78** | **3.89** | 3.50 | 0.39 |
|  | Sept | **20.34** | - | 0.58 | 1.16 | 5.23 | 0.58 | 12.79 | **67.44** | - | 30.23 | 37.21 | **11.05** | 11.05 | - | - | - | **-** | **-** | **1.16** | 1.16 | - |
|  | Oct | **21.32** | - | - | 9.84 | 3.28 | - | 8.20 | **57.38** | 1.64 | 31.15 | 24.59 | **19.67** | 19.67 | - | - | - | **-** | **-** | **1.64** | 1.64 | - |
| **IRI** | Jun | **883.06** | - | 0.39 | 2.48 | 42.70 | - | 575.14 | **2904.41** | 0.35 | 634.09 | 814.56 | **13865.05** | - | - | - | 13865.05 | **-** | **-** | **6.61** | 1.12 | 2.19 |
|  | Jul | **7124.12** | - | 30.82 | 1357.29 | 10.54 | - | 2391.35 | **5400.23** | - | 2986.80 | 336.83 | **1731.76** | 1731.76 | - | - | - | **-** | **-** | **36.95** | 12.51 | 6.06 |
|  | Aug | **4757.95** | 37.03 | - | 207.16 | 1.20 | 1.37 | 2456.48 | **1654.91** | 4.21 | 607.58 | 190.43 | **3860.98** | 2740.16 | 218.68 | 3.95 | - | **1.29** | **5.14** | **323.69** | 264.19 | 2.94 |
|  | Sept | **2895.12** | - | 5.07 | 23.40 | 412.82 | 1.96 | 526.23 | **11902.08** | - | 2407.13 | 3548.75 | **298.96** | 298.96 | - | - | - | **-** | **-** | **10.86** | 10.86 | - |
|  | Oct | **1604.74** | - | - | 132.43 | 136.71 | - | 378.72 | **9763.73** | 8.77 | 3195.82 | 1299.17 | **617.88** | 617.88 | - | - | - | **-** | **-** | **16.86** | 16.86 | - |

F = "frequency of occurrence", IRI = "index of relative importance", N = "numerical percentage of prey", n = "number of prey organisms", and W = "weight percentage of prey.
