# Supplementary material for: Getting What Is Served? Feeding Ecology Influencing Parasite-Host Interactions in Invasive Round Goby Neogobius melanostomus
Source: PLoS One. 2014 Oct 22;9(10):e109971. doi: 10.1371/journal.pone.0109971 (PMC4206283; doi:10.1371/journal.pone.0109971)
Supplement: Text S1 — Size measurements and condition factors of N. melanostomus . (DOCX) [file pone.0109971.s006.docx]

**Text S1**

**Size measurements and condition factors of *N. melanostomus***

Total lengths, total weights and condition factors of *N. melanostomus* were significantly smaller in the Rhine than in the Main. Total lengths ranged from 6.3 cm to 15.3 cm (mean ± SD = 10.0 ± 1.8 cm) in the Main and from 6.1 cm to 14.8 cm (mean 9.5 ± 1.7 cm) in the Rhine (*t*-test; *t*= -3.056, *p*= 0.002). Total weight ranged from 3.914 g to 59.075 g (17.741 ± 10.680 g) in fishes caught in the Main, and from 3.339 g to 50.459 g (mean 13.715 ± 8.898 g) in the river Rhine (*t*= -3.808, *p*= 0.0002; for monthly means see Table S1). Mean condition factors (CF [g*mm^-3^]) of gobies ranged from 1.03 to 2.16 (mean 1.47 ± 0.20) in the Main and from 0.94 to 2.38 (mean 1.39 ± 0.21) in the Rhine (*t*= -3.440, *p*= 0.0007). Length-weight regression analysis revealed a marginally greater slope (m) for the Rhine (m = 3.15, R² = 0.96) than for the Main (m = 3.05, R² = 0.97). In both rivers, the sex ratio was balanced (both samplings comprised 88 males and 87 females).
