# Supplementary material for: Getting What Is Served? Feeding Ecology Influencing Parasite-Host Interactions in Invasive Round Goby Neogobius melanostomus
Source: PLoS One. 2014 Oct 22;9(10):e109971. doi: 10.1371/journal.pone.0109971 (PMC4206283; doi:10.1371/journal.pone.0109971)
Supplement: Text S2 — Genetic identification of parasites. (DOCX) [file pone.0109971.s007.docx]

**Text S2**

**Genetic identification of parasites**

Genomic DNA was isolated and purified from individual parasites using a genomic Tissue DNA extraction kit (Peqlab Biotechnology GmbH, Erlangen, Germany) according to the instructions of the manufacturer. The rDNA region comprising the ITS-1, 5.8S, ITS-2 and flanking regions (ITS+) was amplified using primers BD1 (5’- GTC-GTA-ACA-AGG-TTT-CCG-TA-3’) and BD2 (5’- TAT-GCT-TAA-ATT-CAG-CGG-GT-3’) [1]. PCR-reaction (50 μl) included 25μl Master-Mix (Peqlab Biotechnology GmbH, Erlangen, Germany) containing dNTP, MgCl_2_, Buffer and Taq-Polymerase, 3 μl of each primer (10 pmol*µl^-1^), 14ddH_2_O and 5 μl genomic DNA. Each PCR reaction was performed in a thermocycler (Eppendorf, Germany) under the following conditions: after an initial denaturation at 94ºC for 120 sec, 40 cycles of 94ºC for 20 sec (denaturation), 51ºC for 20 sec (annealing), 72ºC for 50 sec (extension), followed by a final extension at 72ºC for 5 min. Samples without DNA were included in each PCR run. PCR products were examined on 1% agarose gels. A 100bp ladder marker (peqlab Biotechnology GmbH, Erlangen, Germany) was used to estimate the size of the PCR products. To prepare the samples for the sequencing, PCR products were purified with a Cycle-Pure Kit (Peqlab Biotechnology GmbH, Erlangen, Germany). Afterwards the PCR products were sequenced by Seqlab Sequence Laboratories (Goettingen, Germany) using primer BD1 (5’- GTC-GTA-ACA-AGG-TTT-CCG-TA-3’). For species identification, the obtained sequences were compared with Genbank data using the BLASTn algorithm [2].

**References**

1. Král’ová-Hromadová I, Tietz DF, Shinn AP, Špakulová M (2003) ITS rDNA sequences of *Pomphorhynchus laevis* (Zoega in Müller, 1776) and *P. lucyi* Williams & Rogers, 1984 (Acanthocephala:Palaeacanthocephala). *Systematic Parasitology*, 56: 141-145.
2. Altschul SF, Gish W, Myers WMEW, Lipmann DJ (1990) Basic local alignment search tool. J Mol Biol, 215: 403-410.
