# Supplementary material for: Getting What Is Served? Feeding Ecology Influencing Parasite-Host Interactions in Invasive Round Goby Neogobius melanostomus
Source: PLoS One. 2014 Oct 22;9(10):e109971. doi: 10.1371/journal.pone.0109971 (PMC4206283; doi:10.1371/journal.pone.0109971)
Supplement: Text S3 — Size measurements of D. villosus and E. trichiatus. (DOCX) [file pone.0109971.s008.docx]

**Text S3**

**Size measurements of *D. villosus and E. trichiatus***

Analysis of adult *D. villosus* revealed significantly larger and heavier females in the Main, with mean lengths of 11.07 ± 2.06 mm compared to 9.84 ± 1.81 mm in the Rhine (*t*-test; *t*= -3.930, *p*< 0.001) and a mean weight of 0.031 ± 0.017 g compared to 0.020 ± 0.012 g in the Rhine (*t*-test; *t*= -5.085, *p*< 0.001; Figure S1). Similarly, *E. trichiatus* females were larger in the Main (9.70 ± 1.86 mm) than in the Rhine (9.20 ± 1.37 mm; *t*= -2.184, *p*= 0.03) but were not heavier in the Main (0.018 ± 0.012 g) than in the Rhine (0.016 ± 0.009 g; *t*= -1.402, *p*= 0.16; Figure S1). No significant difference was detected in males of both species (*t*-tests; *t*= -0.370, *p*≥ 0.067).
